# Ionizing Radiation Measurement Solution in a Hospital Environment

**DOI:** 10.3390/s18020510

**Published:** 2018-02-08

**Authors:** Antonio-Javier Garcia-Sanchez, Enrique Angel Garcia Angosto, Pedro Antonio Moreno Riquelme, Alfredo Serna Berna, David Ramos-Amores

**Affiliations:** 1 Department of Information and Communication Technologies, Universidad Politécnica de Cartagena (UPCT), Campus Muralla del Mar, E-30202 Cartagena, Spain; pamr0@alu.upct.es; 2 Services, General Electric Healthcare España S.L.U., 28023 Madrid, Spain; enrique.garciaangosto@ge.com; 3 Department of Radiophysics, Hospital General Universitario Santa Lucía, C/Mezquita, s/n, Paraje Los Arcos, E-30202 Cartagena, Spain; alfredo.serna@carm.es (A.S.B.); david.ramos@carm.es (D.R.-A.)

**Keywords:** gamma radiation, healthcare workers safety, dosimetry solution, verification, evaluation

## Abstract

Ionizing radiation is one of the main risks affecting healthcare workers and patients worldwide. Special attention has to be paid to medical staff in the vicinity of radiological equipment or patients undergoing radioisotope procedures. To measure radiation values, traditional area meters are strategically placed in hospitals and personal dosimeters are worn by workers. However, important drawbacks inherent to these systems in terms of cost, detection precision, real time data processing, flexibility, and so on, have been detected and carefully detailed. To overcome these inconveniences, a low cost, open-source, portable radiation measurement system is proposed. The goal is to deploy devices integrating a commercial Geiger-Muller (GM) detector to capture radiation doses in real time and to wirelessly dispatch them to a remote database where the radiation values are stored. Medical staff will be able to check the accumulated doses first hand, as well as other statistics related to radiation by means of a smartphone application. Finally, the device is certified by an accredited calibration center, to later validate the entire system in a hospital environment.

## 1. Introduction

The exposure of healthcare workers to ionizing radiation is a relevant concern due to the health risks involved [1]. Governments and authorities are working to foster ionizing radiation safety. In this way, recent directives (e.g., Council Directive 2013/59/Euratom) [2] have set basic safety standards to protect the health of individuals from dose exposures. Under these directives, healthcare workers in charge of radiology exposed to this type of radiation need to be monitored on a daily basis.

The most effective way of protecting medical staff who regularly work in controlled areas is the continuous measurement of the accumulated radiation level in their bodies. To this end, we have performed an exhaustive analysis of the current commercial radiation monitoring instruments, and divided them into two main groups: personal dosimeters and area survey meters. Personal dosimeters are small devices which healthcare workers wear on the body part most exposed to radiation. Thermo-luminescence based solutions are the most widely used technology where, after a period (usually a few weeks), the device is sent to a specialized laboratory to analyze the received doses. Therefore, the measurement is off-line, which restricts its usefulness. On the other hand, area dosimeters measure the radiation doses in real time, with most of them lacking Internet connections or database storage. These large devices are only placed in certain areas of the hospital (for instance, in the Nuclear Medicine department).

To overcome these drawbacks, a system has been designed, developed, verified and checked as a hybrid solution, valid as both a personal dosimeter for specific zones and an area dose meter. The entire system is based on open-source technology, integrating commercial off-the-shelf (COTS) electronic components including Internet connections via 3G, Ethernet or WiFi. The system functionality is the following one: a device collects the doses employing a commercial GM tube and dispatches them to a remote database hosted in a server. It is responsible for storing all the information received for each healthcare worker, immediately calculating accumulated, average and deviation doses, and providing a highly flexible solution. Also, medical staff are aware of these values in real time, thanks to a smartphone application. This code, as well as the remaining software developed for this work, is available in [3].

The device was verified at the National Dosimetry Centre (CND) in Valencia, Spain under reference laboratory conditions. The complete solution was tested at the Sta. Lucia University Hospital, located in Cartagena (southeastern Spain), using two devices. The first one was placed in a corridor next to the PET department and the second one at the control area of the Computer Tomography (CT) area. The results were compared to calibrated electronic dosimeters, demonstrating the correct procedure carried out by the system’s measurement.

The rest of this paper is organized as follows: Section 2 introduces the technological background related to this research, where dosimetry concepts and a commercial dosimeter classification are thoroughly described; Then, the following Section 3 presents our proposal detailing both its hardware and software architecture, thus, the benefits of the solution offered in respect to the remaining commercial dosimeters are also highlighted. Section 4 deals with the procedure for verifying the device in hospital environments; Section 5 validates our system at Sta. Lucia University Hospital; results are analyzed and discussed, and finally, Section 6 summarizes the main conclusions.

## 2. Technological Background

### 2.1. Ionizing Radiation

Ionizing radiation is that with enough energy to ionize matter, by removing electrons from their states joined to their corresponding atom. It may be produced by radioactive substances, which emit such radiation naturally, or by artificial generators such as X-ray generators and particle accelerators.

The ionizing radiation sources naturally occurring in the earth’s surface can be classified as alpha, beta, gamma or X-ray particles. Their features are explained in the following paragraphs:

- Alpha particles (α): They are usually generated in nuclear reactions or radioactive disintegration. Their capacity of penetration is low. They quickly lose their kinetic energy in the atmosphere, because they interact strongly with other molecules due to their great mass and electrical charge, generating a significant number of ions per centimeter of covered length. In general, they cannot pass through the thickness of multiple sheets of paper. However, if an alpha emitter enters the body it can be very harmful.
- Beta particles (β): They have greater penetration than alpha particles. They can be stopped by a few centimeters of wood or a thin sheet of metal. However, as with alpha particles, they can cause serious damage to the human body.
- Gamma particles (γ): Gamma radiation (γ) is a type of electromagnetic radiation [4]. Gamma rays can cause serious damage to the nucleus of cells, so they are used to sterilize medical equipment and food. Gamma rays easily pass through the skin and other organic substances; therefore, they could cause serious damage to internal human organs. X-rays are included under this category. They are also photons, but with a lower penetration capacity than gamma rays.

### 2.2. Recommended Criteria to Assign Dosimeters for Healthcare Workers

- The Spanish Health Protection regulation against Ionizing Radiation (Royal Decree 783/2001 (BOE 26/07/2001) defines exposed healthcare workers as those who, due to the circumstances in which their work is carried out, are habitually/occasionally subject to the risk of exposure to ionizing radiation that may involve doses higher than the limits recommended for members of the public. Exposed healthcare workers are further classified into two categories, A and B:
  1. Category A includes those who can receive an effective dose greater than 6 mSv (miliSievert) per official year, or a dose greater than 3/10 of the equivalent dose limit for eye lenses (45 mSv/year), skin and limbs (150 mSv/year). In Category A, exposed workers must use a personal dosimeter.
  2. Category B includes exposed workers who rarely receive effective doses in excess of 6 mSv per official year, or 3/10 of the equivalent dose limit for eye lenses, skin and limbs. These workers are not necessarily required to wear personal dosimeters. It is sufficient for them to be controlled by a dosimetry monitoring system that guarantees that the doses received are compatible with their level B classification.
  3. Non-exposed workers, such as members of the public health system, do not require any dosimetry control.

Table 1 summarizes the effective dose levels together with the equivalent doses in eye lenses, skin and limbs used in this staff classification.

A minimum number of people working under ionizing radiation conditions in the healthcare environment are classified as Category A exposed workers, and would be required to be controlled by individual dosimetry. These workers are the following:

- Those who have to stay close to the radiation beam in interventional radiology and hemodynamics.
- Healthcare workers preparing and administering radioactive doses in nuclear medicine.
- Healthcare workers who are involved in the preparation of treatment and care of patients in radioactive metabolic therapy.
- Healthcare workers performing these functions in brachytherapy.

Table 2 shows the percentages of workers with accumulated annual doses exceeding the annual dose limit for members of the public (1 mSv per year, established by the General Radiation Protection Manual, CSN/SEPR/SEFM Forum, June 2003), classified by category and work type.

### 2.3. Dosimetry Classification

#### 2.3.1. Personal Dosimetry

Dosimetry estimates the doses received by a given person. Depending on the body area you wish to estimate, personal dosimetry is classified into:

1. Dosimetry of the whole organism: It is implemented by a lapel dosimeter, which must be placed in a position that is representative of the most exposed part of the body surface. In those cases where a lead apron is required, the dosimeter should be placed under the apron.
2. Dosimetry of the upper extremities: It is implemented by wearing wrist or ring dosimeters. Recommended only in those cases where the doses to the hands can be notably higher than those of the whole organism, due to their proximity to the radiation beam.
3. Abdomen dosimetry: It is implemented by lapel dosimeters. Only used on the abdomen of exposed pregnant workers. Where a lead apron is required, the dosimeter should be placed under the apron.

There are a lot of personal dosimeters on the market. The most common ones are described below:

● Thermo-luminescence dosimeter (TLD) [5].

It incorporates anodized aluminum foil with four thermo-luminescent detectors. These detectors are usually made of lithium fluoride activated with magnesium or calcium fluoride activated, in turn, with manganese.

The detectors store the energy received from ionizing radiation. In order to know the amount of radiation received by the dosimeter, it is necessary to heat it to a temperature of 300 °C, thus releasing the stored energy in the form of light. The amount of light emitted is proportional to the radiation dose received by each detector.

The lapel dosimeter is designed for the measurement of deep personal equivalent doses denoted as Hp (10) and superficial doses called Hp (0.07) [6], as well as for energy discrimination from incident radiation.

The main advantages are its low cost, easy handling, sensitivity, and that it does not depend only on environmental conditions. Furthermore, it is reusable: Once the dosimeter receives the radiation dose during a period of time, it can be employed again. However, its main drawback is related to that the radiation cannot be observed in real time, which negatively impacts on its applicability.

● Film dosimeter [7].

It contains a small piece of radiographic film, placed between two metal filters (usually aluminum or copper) inside a plastic casing, in order to protect the film from light exposure. Some film dosimeters have two emulsions, one for low doses and the other for high dose measurements. These two emulsions can be on separate film substrates or on either side of a single substrate.

When the film is irradiated, an image of the protective box is projected onto the film. In this way, the amount of radiation received is proportional to the optical density of the film being exposed, compared to the optical density of previously calibrated films.

The main advantage is its functionality. Film can provide information on the exposure conditions such as the direction of the incident radiation or suspected contamination. The main drawback is that the reading is estimated, not immediate, and not reusable. It also depends on external conditions, such as humidity.

● Radiophotoluminescent Glass Dosimeter (RPLGD) [8].

It consists of a housing that incorporates a radiophotoluminescent (RPL) glass in the center. The RPL crystal material is activated silver with crystallized phosphate glass (P_2_O_5_). When this material is exposed to radiation, stable luminescent centers are created in the silver ions, denoted as Ag° and Ag^2+^. These ions emit light when stimulated. To produce this stimulation, the crystal is irradiated with ultraviolet light, measuring the light emitted with a photo multiplier tube. So, the intensity of the light emitted will be proportional to the radiation dose received.

The main advantage is its reusability. In the reading procedure, the stored dose is not eliminated. Therefore, it allows us to accumulate the radiation dose in the very long term. In addition, it is not sensitive to ambient temperature, which favors the measurement precision. The main drawback is that it doesn’t measure data in real time and it doesn’t export data.

● OSL (Optically stimulated luminescence) dosimeter [9].

These dosimeters incorporate a sheet of carbon-activated aluminum oxide, located between filters, to obtain qualitative information on the conditions during exposure. To know the amount of radiation dose received, the aluminum oxide is stimulated at specific frequencies by means of a laser. In this way, the intensity of the light emitted will be proportional to the dose of radiation received.

As in previous cases, its main advantage is its reusability. It can be reused a few number of times without losing sensitivity. On the other hand, commercial devices lack remote connectivity and data storage.

● Electronic Personal dosimeter [10].

It uses electronic sensors and signal processing, and shows the radiation dose normally received in microSievert (µSv). This dosimeter either has a miniature Geiger-Müller tube or is equipped with silicone sensors.

The main advantage is that it can continuously display the accumulated dose on the screen, so it is often used as an alternative in emergency cases in which it is necessary to know a specific amount of radiation within a short period of time. Additionally, it can incorporate alarms if a previously programmed accumulated radiation dose is exceeded during a certain period of time. It works with batteries. The main drawbacks are the cost and the lack of connectivity.

● Pen/pocket dosimeter [11].

It is a small ionization chamber, cylindrical (approximately 2 cm^3^) in shape, filled with air and equipped with a central electrode. It is shaped like a ballpoint pen and contains an ionization chamber with a quartz fiber electrode that functions as an electroscope. By means of an optical arrangement, the response of the radiation action can be observed.

The main advantage of this equipment is that the reading of this dosimeter is straightforward and very easy to use. The main drawback is the fact that it has a poor range of use and low sensitivity compared to other dosimeter systems. It requires daily radiation monitoring and daily charging. Occasionally, it can even be discharged when hit. Therefore, for some years now, its use has been reduced significantly.

Finally, Table 3 summarizes the radiation ranges for each personal dosimeter type listed above.

#### 2.3.2. Non-Personal Dosimetry

Non-Personal Dosimeters are devices that estimate the received dose in a particular place [12]. They can be classified as:

1. Area dosimetry: It is performed by means of lapel dosimeters. It is reserved for the estimation of doses in places or areas where workers are exposed under category A.
2. Dosimetry of rotating workplaces: It is performed using lapel and/or wrist dosimeters. It is focused on estimating doses in those workplaces where an activity is occasionally performed, and category B workers could be exposed.
3. Research dosimetry: It is carried out using lapel and/or wrist dosimeters. It is requested by a Radiation Protection Service to measure doses in all those situations not covered in the previous cases.

The following paragraphs will describe the main types of area dosimeters used in hospital environments today. To this respect, they can be divided into two main groups: (i) gas detectors and (ii) solid-state detectors.

● Gas detectors

Gas detectors are based on the direct gathering of the ionization phenomenon caused by a particle when passing through a noble gas, enclosed between two electrodes subjected to a potential difference. This type of gas is used because the dose rate that can be monitored should be as high as possible. In this group, there are three main types of detectors: (1) ionization chambers; (2) proportional counters and (3) Geiger-Müller counters. Each one accomplishes specific functions, implying different shapes and sizes.

1. Ionization chamber [13]. It is the most straightforward gas detector, considered as a plane-parallel capacitor, in which the region between planes is completely filled with a gas, preferably with air. The electric field in this region prevents the recombination of the ions with electrons, which involves electrons moving towards the positive electrode, while positively charged ions move to the negative electrode. The applied voltage sets the velocity at which electrons and ions move to the chamber electrodes. For instance, for a typical voltage value of about 100 V, ions move at speeds of 1 m/s. Under these conditions, ions take up to 0.01 s to get through a 1 cm thick chamber (note that electrons are more mobile than ions and they will move 1000 times faster). An inconvenience is that these temporal values are excessively long for the time dealt with in radiation detection (e.g., a Computer Tomography).
2. Proportional counter [14]. It consists of a metal case filled with a noble gas such as argon or xenon, with a very fine thread crossing the center. The wire (anode) is set at a high potential difference in relation to the box, so that an electric field constantly passes through the gas. The process is the following: when an ionizing particle, such as an electron, goes through the gas, it releases electrons from its atoms (it ionizes the atoms), leaving behind a positively charged ion and a free electron. The free electrons generated in the gas are accelerated to the anode and their amount is proportional to the initial energy of the particle or X-ray. Unlike simple ionization chambers, the potential difference used in proportional counters is much greater, so the electrons accelerated towards the anode have enough energy to produce secondary but proportional ionizations (hence the name of the device), causing an electronic “cascade”. The electric current or voltage generated in the anode can then be measured and digitized and, as mentioned above, the voltage or charge is proportional to the energy of the particle or X-ray incident. To be able to observe individual pulses, we must increase the applied voltage (which means exceeding 1000 V, its main inconvenience). To this respect, the largest electric field is capable of accelerating electrons so that they can generate secondary ionizations. Accelerated secondary electrons produce new ionizations, eventually creating an avalanche or cascade of ionizations.
3. Geiger-Müller counter [15]: If the electric field increases further, the Geiger-Müller region is reached. In this case, secondary avalanches are generated anywhere in the tube, caused by photons emitted by atoms stimulated in the original avalanche. These photons move relatively far from the original avalanche location and, in a short time, the entire tube is involved in the process. Counters based on this principle are known as Geiger-Müller meters. Since the entire tube is involved in each initial avalanche, there is no information on the energy of the original radiation (all incident radiation produces identical output pulses valued around 1 V). So, Geiger-Müller counters are employed as pulse counters. However, in these counters there is a serious problem. During their movement into the tube, ions can be accelerated, reaching the anode with enough energy to release electrons and starting the process again (due to the nature of the multiple avalanches). To overcome this inconvenience, a second type of gas, called quenching gas, is added. This gas is made up of complex organic molecules such as ethanol (while the primary gas is usually made up of simple molecules, such as argon). A typical mixture of gases will be: 90% argon and 10% ethanol. The molecular nature of this added gas prevents the appearance of continuous avalanches. Geiger-Müller sensors allow the obtaining of accurate radiation doses with a remarkably low cost, and its integration into any electronic system to manage the information and dispatch it to remote places is a straightforward process. These are the main reasons why the Geiger-Müller counter has been adopted as part of our proposal.

● Solid State Detectors

The solid state detector, also known as Semiconductor Radiation Detector, is a radiation detector in which a semiconducting material (silicon or germanium) constitutes the detecting medium. Solid detectors have higher densities that provide reasonable absorption probabilities for a normal detector size. Two types will be described: (1) scintillating counters and (2) semiconductor detectors:

1. Scintillator counters [16]. The scintillator counter works as follows:

- Incident radiation interacts with the atoms and molecules of the material, stimulating them.
- Stimulated states are de-energized by emitting visible (or nearly visible) fluorescent light.
- The light reaches a photosensitive surface by pulling out photoelectrons.
- The electrons accelerate and multiply in number to form an electrical pulse.

There is a wide variety of available scintillators and photomultiplier tubes, depending on the type of application. The properties to be considered in the selection of the material include the fraction of incident energy that appears as light, efficiency (the probability of radiation being absorbed), response time and energy resolution.

2. Semiconductor detectors [17]

Semiconductor solid materials (germanium and silicon) are alternatives to scintillators for building radiation detectors. When a type-p semiconducting material is in contact with a type-n semiconducting material, the electrons of the type-n semiconductor can diffuse through the junction in the type-p semiconductor and combine with the vacant ones. In the vicinity of the p-n junction, the charge conductors are neutralised, creating a region called the depletion zone. The diffusion of electrons from the n-type region leaves behind ionized, fixed donor states, while in the p-type region there are fixed acceptor states charged negatively. Thus, an electric field is created, which ultimately makes diffusion phenomenon advances impossible. A typical p-n junction of a diode is formed.

If any radiation penetrates into the depletion zone and creates an electron-hole pair, the result is very similar to that of an ionization chamber. The electrons flow in one direction and the vacancies/holes in the other. The final number of collected electrons creates an electronic pulse whose amplitude is proportional to the energy of the radiation.

Concerning the manufacturing of these detectors, it is possible to initiate them from a p-type semiconductor in which lithium atoms are diffused. The n-type layer created by producing detectors such as Ge (Li) or Si (Li) is in the order of 1 mm thick, which is easily penetrable by medium-energy gamma rays (the range of a photon of 100 kiloelectronvolt in Ge is about 4 mm and in Si about 2 cm). However, in the case of charged particles the range is much smaller (e.g., for 1 megaelectronvolt electron the range is 1 mm in Si and Ge; for 5 megaelectronvolt alpha particles, the range is only 0.02 mm in both) and there is a 1 mm thick layer to prevent the particles from accessing the depletion zone.

The main advantages are that the time required to collect the load of large detectors is in the range of 10–100 ns, depending on the shape of the detector (flat or coaxial) and the point of entry of the radiation with respect to the electrodes. This time is less than that obtained in an ionization chamber, since here the path followed by the created loads is reduced by several orders of magnitude. Another clear advantage of these types of solid detectors is related to energy. Less energy is required to create an electron-hole pair (~3.6 eV/pair in Si at 300 °K), thus, excellent energy resolution is obtained. An inconvenience is the lack of connectivity in commercial devices.

### 2.4. Discussion

As for personal dosimetry, thermo-luminescent dosimeters are the most widely used in Spanish hospitals. However, as previously mentioned, their main drawback is the lack of real time measurements. Each personal dosimeter will be transferred at least once a month to a specialized center (in Spain, the National Dosimetry Centre—CND) which will heat it to up to 300 °C. The amount of light thus emitted is proportional to the radiation dose received.

During this period of time, in which the CND estimates the level of radiation received by the dosimeter, health personnel will have another dosimeter made available to measure the radiation of the following month. So, a monthly radiation level is obtained and there is no possibility of knowing specific periods of time with higher doses during this time because the radiation dose is accumulated.

As for area dosimetry, most of the devices used in a hospital environment lack any connection to remote systems in order to receive the dose. Usually, the only way to get the data is to be in front of the dosimeter and to check the dose.

On the other hand, there is an important concern regarding data storage. These types of devices store the average radiation values in specific temporal periods. Data are released after each period to restrict data storage. To carry out a later evaluation of emitted radiation, it will not be possible to access radiation values outside of the current period.

In addition, if users want to store the data directly on a PC, it is necessary to have it in the same room as the area dosimeter, which in some cases, can be a complex or impractical task, for instance, in a hospital corridor or in a computer tomography room (cases of study proposed in this work).

In summary, the major drawbacks detected in the current instrumentation for ionizing radiation measurement are described in the following points:

1. Limited storage. There are devices that can store the radiation captured internally. However, due to the limited memory capacity of these devices, it is not possible to have a large amount of stored data.
2. Scarce Flexibility. Most devices do not allow the collection of data in any other way than that established by the manufacturer. In the particular case of personal thermo-luminescent dosimeters, where the stored data can be accessed, radiation collection must be stopped and be replaced by another device while accessing the data.
3. High cost. Those alternatives that have some storage capacity and offer results in accurate, real time radiation doses (especially for area dosimeters) have a clear impact on the cost. Therefore, these solutions are addressed to public entities or big, private companies.
4. Power supply. A large number of dosimeters described in this section are supplied by batteries. In the case of failing batteries, the received dose is not computed and a health risk could result.
5. Manufacturer dependent. A priori, any modification of the device firmware to adapt it to new conditions of the application/environment is not possible.

## 3. Proposal

### 3.1. General Overview

**Figure 1:**
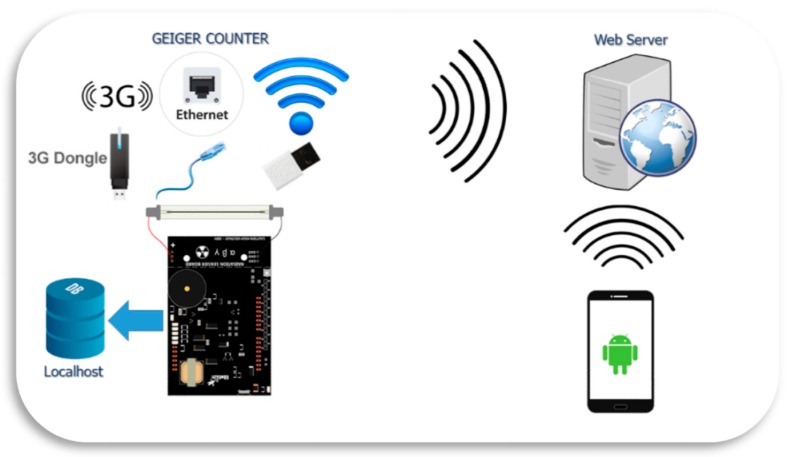
System components.

The radiation measurement proposal is a step forward, which solves diverse problems presented by current radiation systems. Figure 1 shows the main system components where the entire system integrating wireless communications, database, mobile application and measurement device have been highlighted.

As a general approach, the radiation is measured with a commercial Geiger-Müller tube [18], receiving two values: (i) the effective dose-rate value (µSv/h) and (ii) the counts per minute (CPM). These values are stored in two redundant databases: one hosted on a remote web server and the second in a database implemented in a Raspberry Pi [19] (localhost), which in addition, provides memory storage, processing and connectivity. Note that this commercial detector can be replaced by another; without thereby the system functionality lessen. Therefore, it is not an objective of this work to characterize and study this sensor, tackling these concerns in future works. For instance, it should be interesting to model the GM directivity, to later implement it in the Raspberry. Furthermore, in the case of using detectors such as the solid state ones (because the application requires it), environmental parameters (temperature, pressure, humidity, etc.) must be considered to adjust its operation. As in previous case, the detector adjustment will be developed in the corresponding software module of the Raspberry. Under these premises, to have of a COST-based system facilitates the design of specific solutions in accordance with application or service requirements.

The set of radiation measurement, capture, and storage procedure is performed in periods of one second, i.e., the sensor measures radiation doses and counts per minute (both values form the data) at one-second intervals and stores both values in the Raspberry Pi and web server. In this way, it provides a versatile solution for the storage problem that current systems are facing today.

The data uploading to the database of the web server is carried out through 3G, Ethernet or WiFi technology, which ensures flexibility and reliability in the face of any contingency in the transmission of information (e.g., falling 3G network, WiFi router failure, etc.). Furthermore, the user has access to a smartphone application, which is able to show information in real time, or statistics of radiation values computed in remote web server: Daily measurements, averages, deviations and so on.

The user could access data through the web server, as well as being able to export them to other formats, for instance, a spreadsheet (e.g., Excel) to analyze the data in more detail. In addition, access to the data does not stop the operation of the device, which is the main drawback of some of the current systems.

The system could be used either as a personal dosimeter and/or as an area dose meter. Regarding area dosimeters, it is obvious, since its functioning is the same as Geiger counters. To use it as a personal dosimeter, healthcare staff have to log in and register in the smartphone application. So, for each registered user, the received radiation is stored in the corresponding databases, to later consult instantaneous, accumulated, mean radiation values of dose. In this regard, it provides a real-time solution in personal dosimetry, avoiding long waiting periods in order to know the received radiation data, as occurs in many of the current systems (e.g., thermo-luminescent dosimeters) which are connectable from any place through Internet access.

The Geiger-Müller detector is powered by the Raspberry Pi, so this device just has to be connected to the power supply, thus avoiding the energy limitations of other electronic dosimeters. Once connected to the power supply, the first step that the system performs is to connect to the remote database and start receiving radiation values.

Among all the aforementioned technologies, the system has a Geiger-Müller tube, therefore a Geiger counter. A sensor of this type usually triggers pulses, which is not an energy value, so it would not be useful for knowing the dose of radiation received by health personnel. Users could get the radiation value multiplying CPM by a conversion factor (K):

CPM × K (µSv/h)(1)

This conversion factor is achieved by means of a thorough calibration process by the manufacturer. In our case, this factor is valued in 0.008120 [18], thus the radiation received is derived in units of micro Sieverts per hour. The app shows Counts Per Minute (CPM), and Radiation Value (µSv/h) at the same time. This is a new feature that commercial Geiger counters do not provide today.

**Figure 2:**
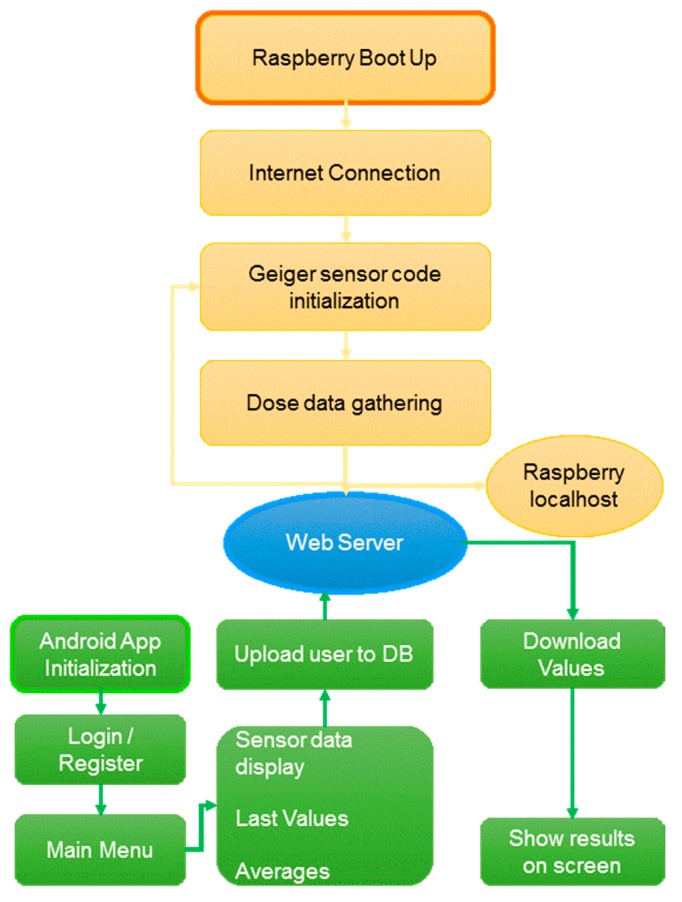
System’s block diagram.

It is important to note that the system is able to estimate the radiation value in real time and send this information to a database. This system contributes to monitoring radiation in two ways: (i) by acquiring/collecting radiation values in real-time and (ii) in the ability to store each radiation value labeling it with its corresponding date/time along with the user receiving the dose. Figure 2 shows how the proposed system works.

Nowadays, there are some alternatives, including GM detectors, which show radiation in real time, and in which data are also stored. To accomplish these functions, in [20], the device only ensures connectivity to a PC via USB, while the system proposed in [21] sets a proprietary system up to send data to a PC. Therefore, comparing with our proposal, the lack of remote communications incurs in a clear disadvantage. Furthermore, an additional advantage to our system, contrasted with similar systems such as the one mentioned above, is its low cost. Currently, this type of system is valued at thousands of Euros. In this sense, the work [22] tests a medical environment through a proprietary and costly solution, although in this case, remote communications are considered. We offer an alternative capable of improving current services at a much lower cost (the prototype proposed here does not exceed 200 Euros).

Different low-cost dosimeter-based solutions can be found in [23,24]. In [23], a dosimeter prototype captures the dose rate at 5 Hz through a CMOS camera. However, this proposal lacks communication support which prevents, for instance, statistics calculations. The work [24] leads to security concerns in power nuclear plant communications when data include radiation values. To face this, authors propose a solution based on XBee module communication, a well-known Wireless Sensor Network (WSN) device. Authors carefully explain aspects such as modulation, packet format or firmware, and how this device takes actions when malicious attacks occur; although they never evaluate its operation under the proposed nuclear environment.

The work [25] presented a low-cost, open-source and remote communication solution. In this paper, authors analyzed the variation of the energy in relation to the dose rate in a commercial and personal X-rays detector denoted as POKEGA. It is conceived to monitor the radiation received by members working in interventional radiology. The POKEGA solution was proposed in other works [26,27]; it is composed of a photodiode detector capturing the radiation, and a smartphone responsible for dispatching the dose (together with, for instance, GPS position, time, date, etc.) to Google docs, to later visualize the emplacement where each dose was collected. Both, photodiode and smartphone are connected by a cable. In comparison with our system providing reliability and robustness to the communication thanks to diverse wireless interfaces, POKEGA depends on the smartphone and its telephony connection. Furthermore, our system stores the monitored radiation both in a remote server and in the local device (Raspberry).

In summary, our system could be used as a personal and area dosimeter. It provides the advantages of displaying data in real time and storing it, either at an area level or for a specific user. In addition, the data reading procedure is more suitable and quicker than in the case of current personal dosimeters.

These features are also ensured by its connectivity (USB, Ethernet, WiFi or 3G). So, this heterogeneous connectivity allows us to know the amount of received doses outside of the measurement location. It is notable that its connectivity, together with the ability to connect it to electrical current, guarantees robustness and reliability. Finally, the device has been verified by the CND simulating hospital conditions and validated in a real hospital environment, giving accuracy to our proposal as a real alternative.

### 3.2. Advantages of Our Proposal in Respect to Current α, β, γ Radiation Meters

The main advantages of our proposed system in respect to other proposals are the following:

1. Low cost. In comparison to other systems, the features and functions offered by our system are more competitive in terms of cost.
2. Double functionality. The device can be used for both personal (in specific places as, for instance, the CT room) and area dosimetry.
3. Real time doses. This system is capable of displaying the dose values in real time either as a personal or area dosimeter.
4. High storage capacity. In contrast to other current systems, our system has a large storage capacity, due to the database usage.
5. Statistics. Several statistics are calculated immediately by the remote server, available for users at any time.
6. Connection alternatives. The device is able to connect to remote databases through a set of interfaces (USB, Ethernet, WiFi and 3G).
7. Data recovery. If the device loses the connection to the remote database, all radiation values are stored in the device until the connection is restored.
8. Flexibility and modulation. The system is built using general-purpose and open-source technologies, which allows software/hardware modifications.
9. Easy access. Users can check the data everywhere, due to the fact that the interface is a smartphone app that is connected to the remote database.
10. Reliability. Different connection alternatives, replicated databases, and continuous energy supply contribute to a robust and reliable system.
11. Verification. The device has been verified in CND, under standard calibration protocols.
12. Easy handling and installation. Once the power is turned on, the device is automatically set to capture radiation, store it on the system and connect to the remote web service.
13. Authentication. Security tasks will be implemented in a future work; however, the user must register to access dose values.
14. Validation. This proposal has been validated at St. Lucia University Hospital in different environments, which will be further described in Section 5.

Finally, Table 4 summarizes a comparison among different current radiation meters. This comparison is extended to include our proposal.

### 3.3. In Detail: Description HW/SW of the System

In the following sections, the system proposed here will be discussed in depth from a twofold perspective: (i) hardware components used, their connection/integration and (ii) software implemented.

#### 3.3.1. Hardware Description

The device hardware is built as the integration of different printed circuit boards. These include:

1. A Raspberry Pi.
2. The radiation sensor board.
3. The Bridge board that connects the Geiger counter to the Raspberry Pi, denoted as Arduino Shield Connection Bridge.

**Figure 3:**
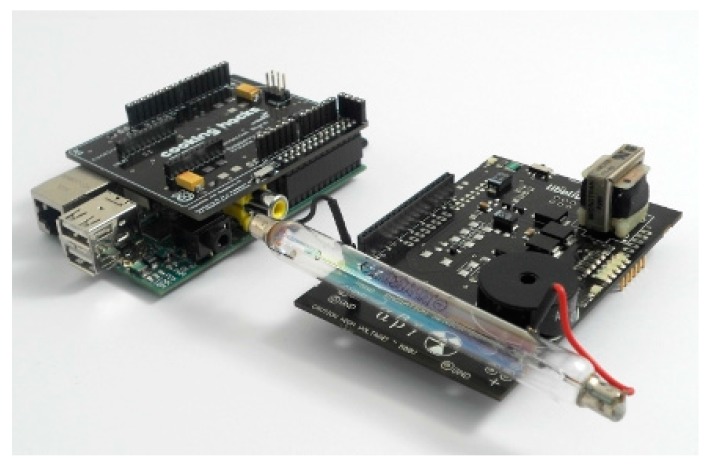
Raspberry Pi and Geiger Sensor (**left**) connected to the Bridge board and the Geiger Sensor board (**right**).

**Figure 4:**
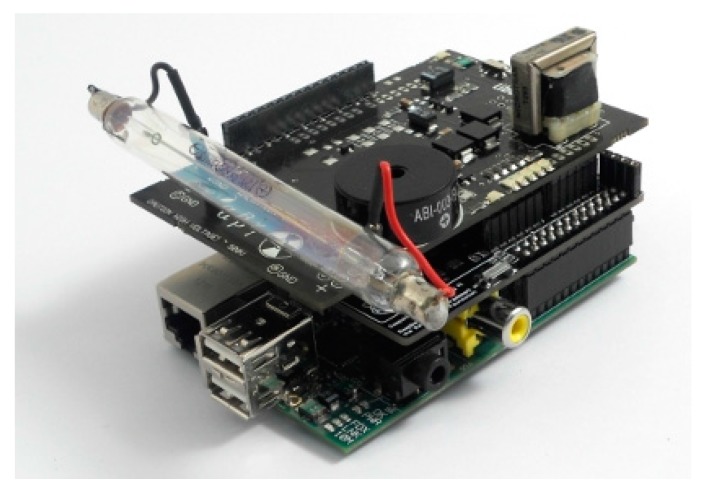
End device.

Figure 3 shows the Raspberry Pi and Geiger Sensor, while Figure 4 depicts the entire system integrated and connected.

In the following paragraphs, we describe the main components of the system in detail.

- Raspberry Pi [19]. It is a low-cost embedded board developed by the Raspberry Pi Foundation in the UK. Its size is approximately that of a credit card and encapsulates a Broadcom BCM2835 chip with an ARM processor up to 1 GHz speed. In our work, the Raspberry Pi B++ model has been selected for its low cost and ability to carry out all the functions of our system. Note that a MicroSD card including the operating system (Raspbian), is inserted in the back of the Raspberry, which will be explained later.

The following components of the Raspberry Pi are employed and configured in our development:

− A/V connector power boards to operate (5 V).
− USB ports used to connect the 3G dongle or wireless adapter. In addition, if the Raspberry needs to be programmed, they will be used to connect the mouse and keyboard.
− A General Purpose Input/Output (GPIO) port consisting of 40 pins allows the Raspberry Pi to communicate with external elements. In particular, GPIO pins are managed and handled directly through code from the intermediate Bridge board.
− An Ethernet port joins our system to the Internet.
− An HDMI port connects the Raspberry to a monitor, for configuration tasks.

The following functions are carried out using the Raspberry:

1. Executing the code corresponding to the Geiger counter in order to display/visualize the dose values.
2. Connecting to the Internet via Ethernet, WiFi adapter or 3G dongle through USB.
3. Enabling the automatic execution of the Geiger counter code and automatic internet connection.
4. Running a localhost, including its corresponding database, which allows us to have a backup database.
5. Ensuring that collected doses are uploaded to the database on the remote web server every second.

- Raspberry Pi to Arduino Shield Connection Bridge [28]. Since the connection between the Geiger Sensor Board and Raspberry is not possible, an intermediate board or bridge is required. This board is called Raspberry Pi to Arduino Shield Connection Bridge, but for the sake of brevity, we will denote it a bridge board.

The goal of this bridge board is to facilitate the integration of modules designed for Arduino on Raspberry Pi. This allows us to attach analog and digital sensors using the same pins for Arduino, but with the power and capacity of a Raspberry Pi. So, on the back of this main board, a bridge board will be connected through the GPIO pins. The board including the Geiger counter will be connected to the pins I/O, UART, SPI and I2C, belonging to the bridge board.

- Geiger Sensor Board [18]. This board is responsible for measuring radiation values using a Geiger-Müller tube. The radiation board is composed of two parts: the supply side and the signal side. The supply side is responsible for ensuring the high voltage required by the Geiger-Müller tube (400 V–800 V). The signal side adapts the output analog pulses from the GM tube to digital data understandable by the Raspberry Pi.

To achieve high voltage in the GM tube, an oscillator connected to a voltage multiplier consisting of diodes, transistors, resistors and capacitors is employed. This electronic circuit guarantees a potential difference of 500 V. In the particular case of requiring voltage values above 500 V to feed the tube, they will be obtained from zener diodes connected in series.

- 3G dongle. When connecting to the Internet via 3G, it is necessary to put a 3G dongle into the interface USB of the Raspberry Pi. Although there are several models compatible with Raspberry Pi, the selected dongle [29] together with a prepaid card have allowed the use of the device anywhere in the hospital environment. In addition, in the specific case of not having access to WiFi or Ethernet in the hospital under study, or when the system was verified at CND, the use of the 3G dongle has been essential to dispatch the captured radiation data to the remote web server.
- WiFi Adapter. This component is placed in a USB port, providing access to the Internet thanks to the WiFi hospital infrastructure. It should be noted that our device ensures an Internet connection (and therefore, the dose information for each user in the remote web server) by selecting of one these technologies: WiFi, 3G and Ethernet.

**Figure 5:**
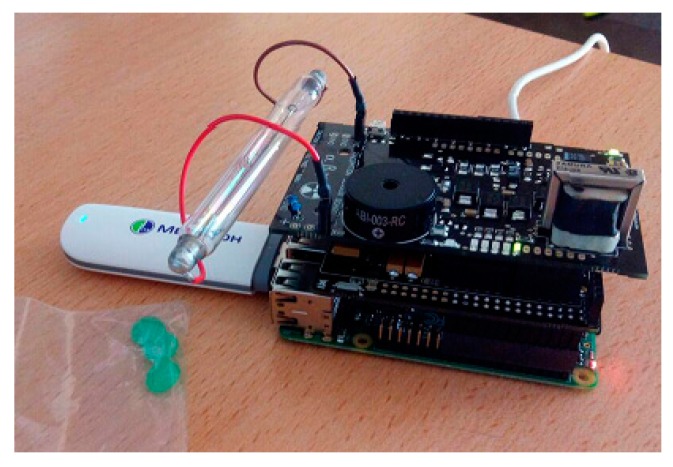
Device with 3G dongle attached.

Figure 5 shows the complete system with 3G USB dongle connected.

**Figure 6:**
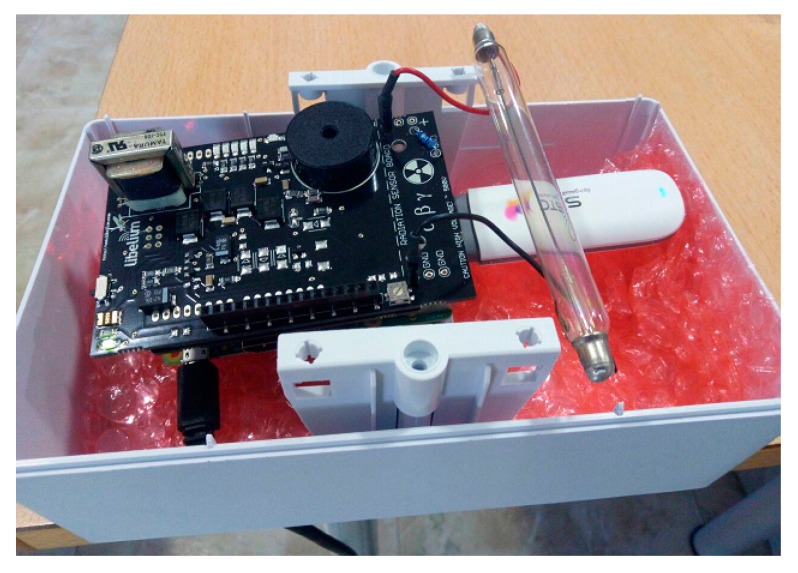
Cover case including device.

Figure 6 depicts the complete system inside the protection cover.

#### 3.3.2. Software Description

1. Operating System. The operating system (OS) for Raspberry Pi is denoted as Raspbian. In particular, Raspbian Jessie was the version installed, because it offers multiple functionality and documentation in terms of capturing data and communication issues.
2. Geiger sensor code. This code, implemented in C language, quantifies the counts per minute and equivalent radiation measured by the GM detector on the board. Raspberry Pi executes the programmed code obtaining the dose data from the Geiger detector through its GPIO pins. Since the sensor uses the same connections as Arduino, it is necessary to include the arduPi library codes provided by the manufacturer.

**Figure 7:**
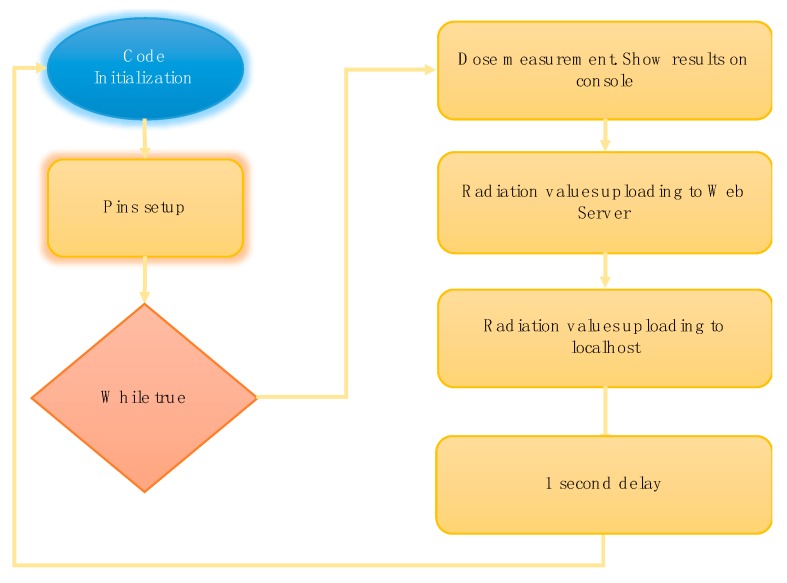
System general diagram.

Figure 7 shows a flowchart of the way a Geiger operates (and its associated code).

Every time the code is initiated, the application remains waiting for a stimulation to occur in the GM tube, increasing the number of counts. Then, the application enters into an infinite loop, in which the radiation values are continuously captured in periods of one second. In reference to the previous figure, each of the blocks is discussed:

● Radiation measurement: display by console terminal

As mentioned above, the app shows counts per minute (CPM) and radiation dose (µSv/h). Firstly, it is necessary to calculate the number of counts per minute and to later obtain the equivalent radiation value related to the CPM calculated, multiplying the CPM by the conversion factor provided by the manufacturer. The number of counts corresponding to the GM tube stimulation is stored in a specific variable. The temporal interval lasts one second. In order to obtain equivalent CPMs, it will be necessary to multiply the number of counts per second by 60 (countPerMinute = 60 ∗ count).

Finally, before the radiation values are stored in the local and remote databases, the number of counts is reset (variable is set to zero), so when the loop is restarted, a new count value is obtained.

● CPM and radiation value upload to remote web server

Once CPM and radiation values have been obtained, the next step is to upload these data to the MySQL remote web server database. To this end, it is necessary to use a method which connects to the server by IP, user name and his/her password. Then, the programmer/developer selects the database which connects to it. Note that each device will have its own database on the web server. Thus, each device is independent of the other ones.

On the other hand, to upload the CPM and radiation values, a SQL query is implemented. In this case, programmers dispatch an INSERT type query, including countPerMinute (CPM) and radiationValue (equivalent radiation) values to the remote database. Finally, once these values have been stored, the connection is closed.

The results displayed by the smartphone application will be the data (together with their statistics) stored in this database installed in the remote web server.

● CPM and radiation value storage in localhost

The dose values are also stored in the localhost database.

Any error detected in the remote web server will be mitigated by the localhost. That is, those radiation values which were lost in the period of non-operation of the web server will be recuperated by the localhost. This function is carried out by a method which shares the same code as the previous one, except for the IP address.

● End of loop

Finally, once data has been uploaded to the web server and stored in the localhost, there is a one second delay in the following set of measurements. This delay implies an appropriate time interval, from the time the radiation level is measured by the Geiger detector, to the time the Android application displays this value on the screen. If the delay decreases to values inferior to one second, the GM detector would be able to measure more accurately. However, there would not be enough time to upload the data to the web server and download it onto the app. It is notable that other systems are capable of showing radiation levels, but taking more time, demonstrating that this system is more accurate in adjusting the intervals to radiation samples.

Finally, an automatic code startup has been implemented. To avoid the manual execution of the code in Raspberry Pi (resulting in inefficient application in hospital environments), a script has been programmed to automatically start the code every time the Raspberry Pi is connected to a power supply or is booted. To this end, as Raspberry Pi is a Unix system, the script will be created within the */etc./init.d* directory, in which the start and stop commands are specified. Additionally, the different interfaces to access Internet (WiFi, 3G and Ethernet) will also connect automatically, without requiring support from specialist personnel or developers.

3. Servers. A server is required to store the dose data collected by the device. The one selected for both the remote web server and the localhost is Apache [30], which is a free-service and open-source HTTP server with the same features as other high-cost competitors. The Apache web server is responsible for storing the GM-captured radiation data and managing them in an Android application. In this case, the Android application accesses this server, both to log in or register a new user, and to display the radiation measurements/statistics. The remote web server requires Internet access, so, it must have a domain and therefore, a public IP address. In a certain way, both the device and the Android application connect to the server through this public IP. Under this premise, note that a web server allows us to access data anywhere. Using the Android application or a PC, we check the radiation values at any time and place, even if the detector fails or turns off. This also allows us to control the login and registration of users, avoiding, for instance, that two different users employ the same user name. The Apache localhost is configured as a backup service, lacks an Internet connection, and data are recovered via USB.
4. Database. They are responsible for storing both radiation data from the GM detector and information from user registers. PhpMyAdmin [31] has been selected as the tool to generate database, both in the local server and the remote web server. PhpMyAdmin written in *PHP* ensures the database management and handles the MySQL facilitates. For instance, the user can create/delete databases, generate/delete/modify tables, delete/edit/add fields, execute any SQL statement, handle keys in fields, manage privileges, and export data in various formats.

Keeping in mind that the devices are independent from each other, labeling each device with its own unique identification number means that each device has its own database. The result is a more intuitive and appropriate management of different devices deployed in a hospital, thus facilitating the data recovery and the data security.

The fields composing the databases are, as well as the CPM and radiation value, the following: (i) an identifier for each measurement uploaded; (ii) a timestamp of the captured dose, both coming from the device; and (iii) registered users, including user name, password and email data, dispatched by the smartphone application. Regarding the latter, the goal is for each hospital to have its own databases of users, which would be independent of the others.

5. Android application. The application developed for smartphones is in charge of displaying all the radiation results captured by devices. This application has been implemented in Android because, among other factors, it is an open source. Furthermore, Android makes the simulation and testing of different versions of our application on different devices easier, without the requirement of them having been previously developed in emulators. The development of the app is divided into two main parts: The appearance, focused on the graphic interfaces and the specific functions of each of them.

**Figure 8:**
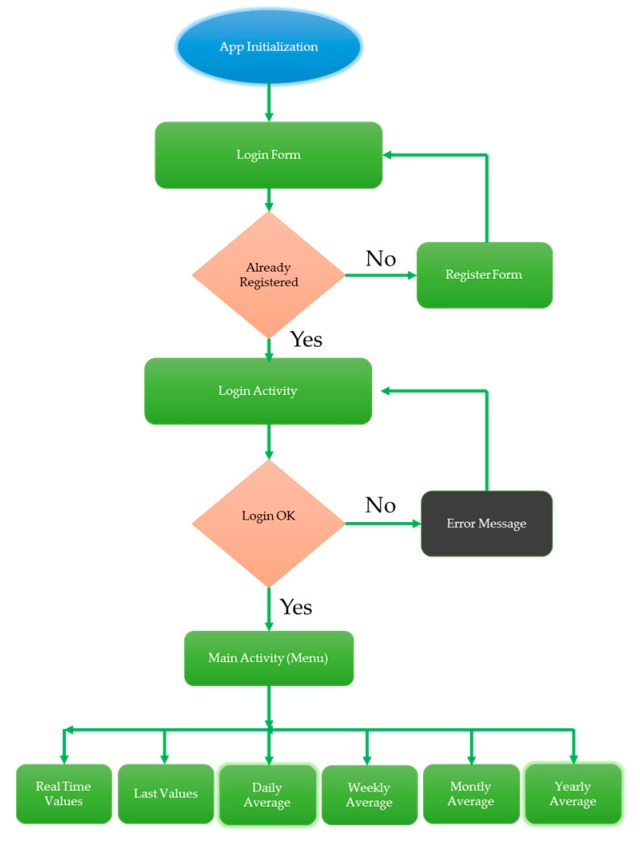
App flow-chart.

Figure 8 presents a flowchart including the overall functionality of the app.

The application links the database through SQL queries. These communications are asynchronous due to the fact that the server runs diverse processes, so this issue will stop the main application if it is coded in a synchronous way. To implement asynchronous tasks, the *AsyncTask* class [32] is employed. This communicates with the process of the main thread, only performing operations and showing the results in the main thread.

An advantage of our implementation is the reduction of the number of database accesses. So, the application is more efficient and dynamic, decreasing the probability of connection errors. To this end, the *Intent* class [33] is in charge of sending the user name and device number, among different activities, without requiring remote connection to the database. Therefore, SQL queries are only demanded when users want to obtain some type of results, such as radiation statistics. Finally, in the following subsections, the appearance and function of each of the parts in which the app has been divided will be detailed, according to the general diagram shown in Figure 8.

##### App Initialization

**Figure 9:**
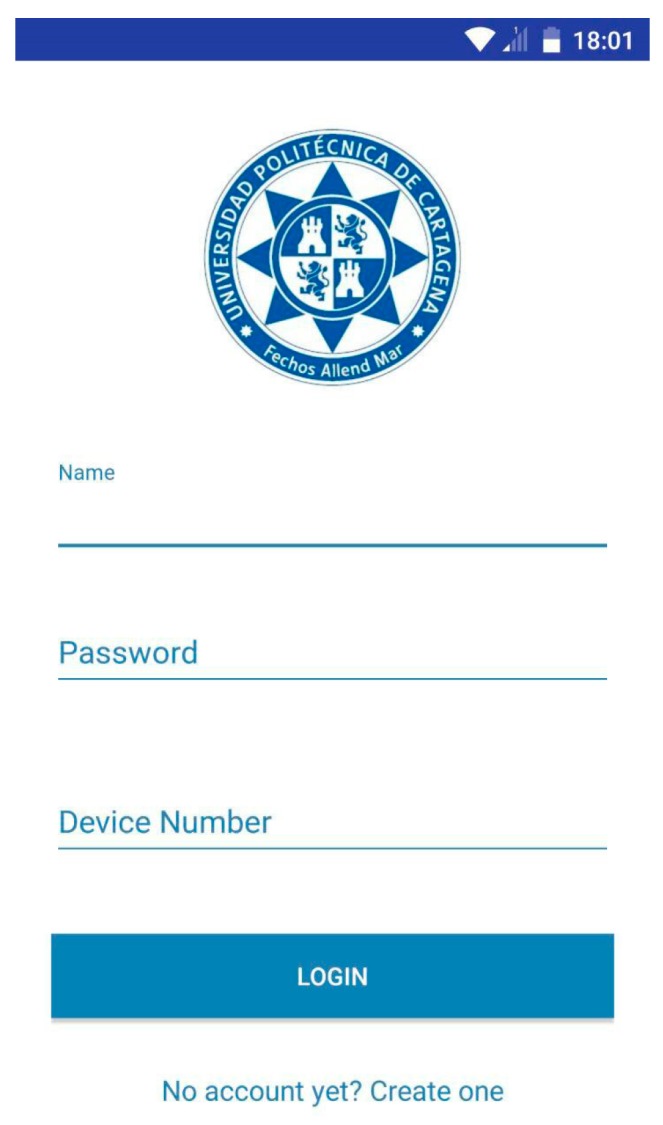
Login screen.

Each time the app is started, an image, like in Figure 9, presenting a login form, appears.

As shown, this form inputs the user name, password and device number. First of all, the user name linked with the password is required. Those values are stored in the database.

The device number is the identifier assigned to each device. Each hospital has a specific number of catalogued devices, allowing the user to select the device which they want to receive radiation information from. This selection is manually accomplished when logging in, and depends on the area where the staff is working. Therefore, by filling in the device number field, the app connects to the database corresponding to that device. This is possible because, as discussed above, each healthcare worker is exposed to radiation in an enclosed hospital area, and it will not be necessary to modify the device number every time.

**Figure 10:**
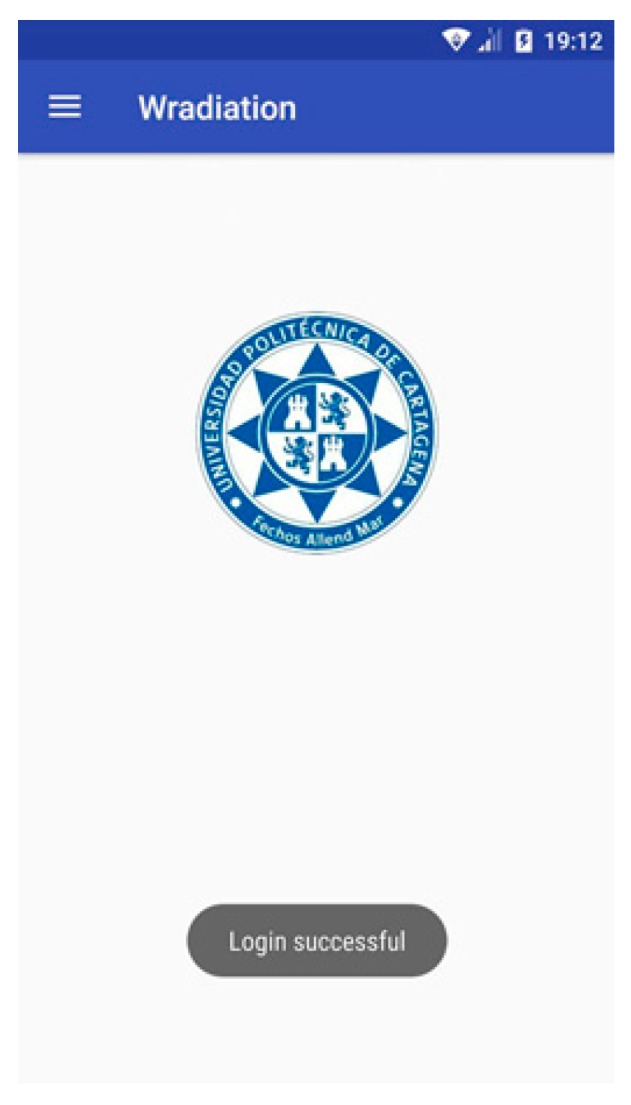
Login successful.

On the other hand, if the user doesn’t input data in the form fields or a device number is not one of those assigned to the hospital, an error message will be shown, alerting the user. The *Inputvalidation* class is in charge of these functions. However, if the proccess is correct, the *Login* button is enabled and the user name and password are checked against the database through the *CheckUser* class. In the affirmative case, the main view of the application is depicted in Figure 10.

**Figure 11:**
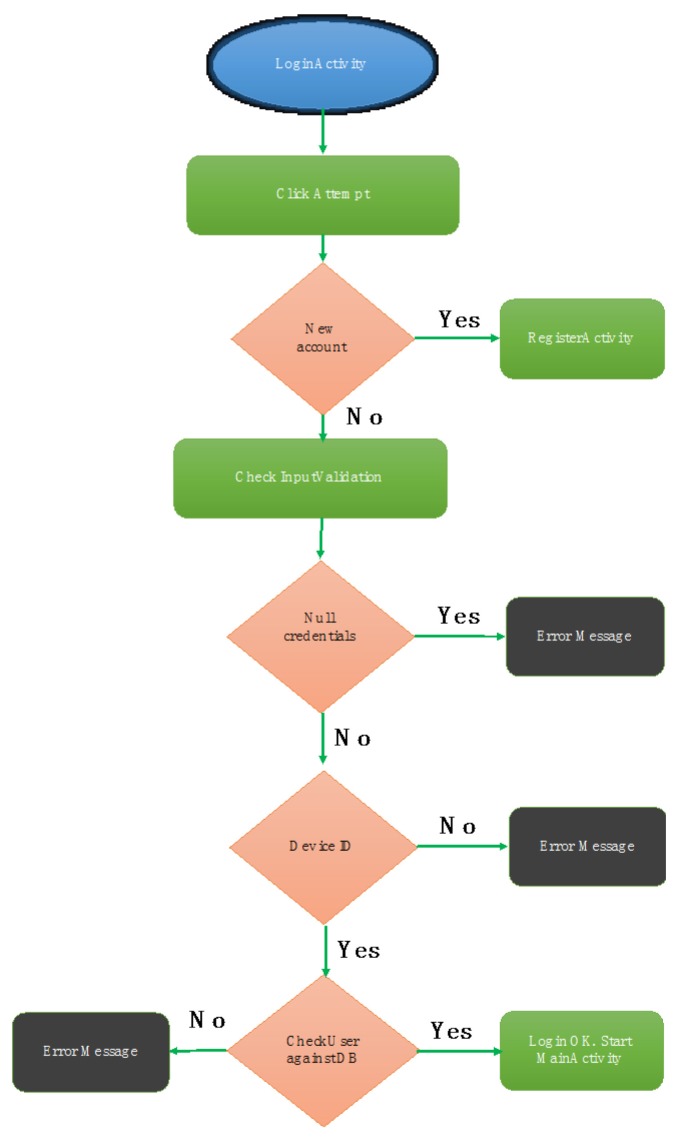
Start procedure flow-chart.

All views and styles have been programmed in XML. To achieve this, the official Android API has been used. The code corresponding to Start session is denoted as “LogicActivity”. The flowchart of this activity is presented in Figure 11.

##### User Register

**Figure 12:**
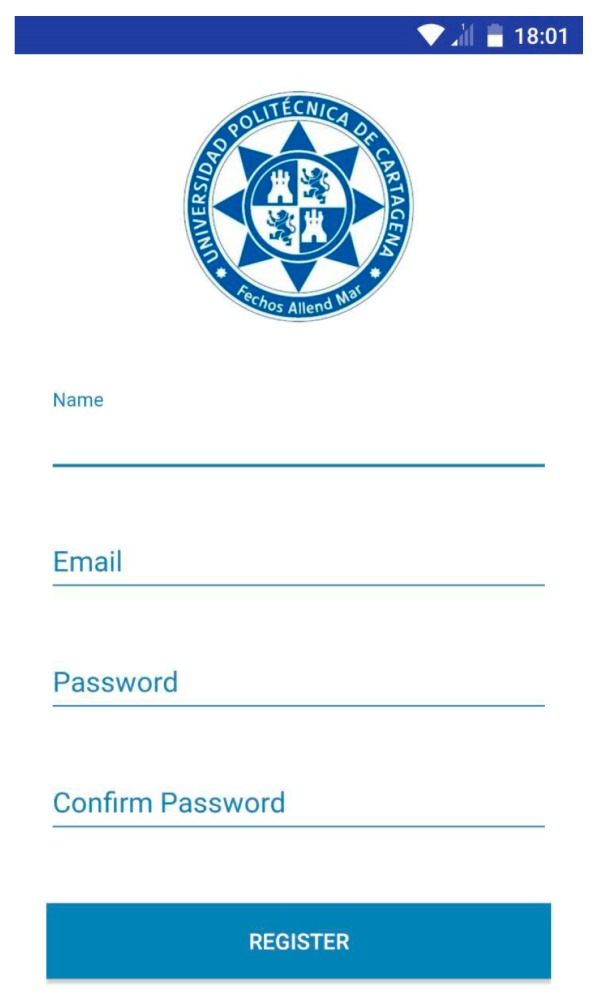
User registration.

Each time the user clicks on the screen “No account yet? Create one”, located in “LoginActivity”, the activity called “RegisterActivity” will start. This activity shows a registration form, as shown in Figure 12.

**Figure 13:**
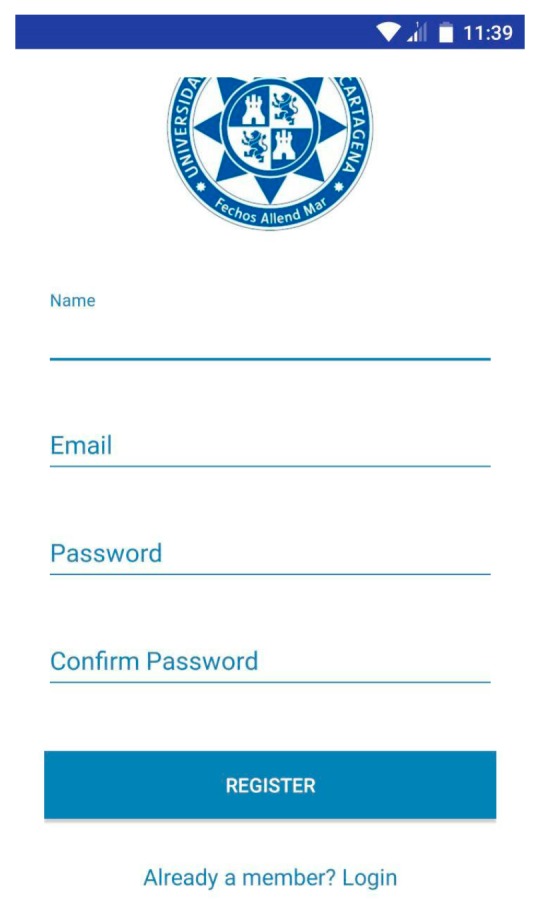
App login screen.

As we can see, this form asks for the user name, e-mail address, password and password confirmation of the user. As in “LoginActivity”, the login form is accessed again by clicking on the sentence “Already a member? Login”, shown in the following Figure 13.

**Figure 14:**
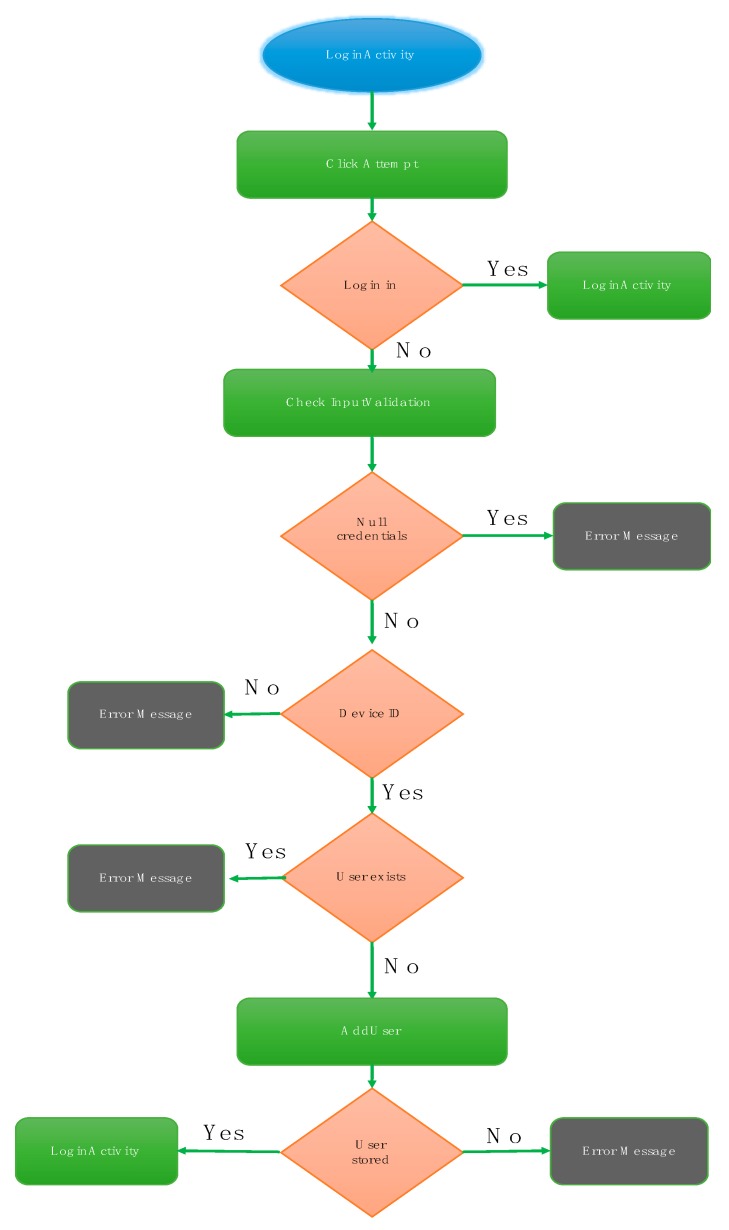
User register flow-chart.

“RegisterActivity” contains the same features as “LoginActivity”. The *InputValidation* class executes a function in charge of ensuring that if the user introduces empty fields in the form or if the *password* field does not match the *confirm password* field, an error message will alert the user. The class *CheckUserRegis*, extended to the class *AsyncTask*, checks if the application has a user name which already exists, thus comparing the one introduced in the application with the user’s database. Finally, if a user name doesn’t match a previous one introduced in the database, it means that this name is available and the new user’s data is stored in the database. The class responsible for this function is called *AddUser*. The following flowchart summarizes how this activity works (Figure 14).

##### Main Menu

**Figure 15:**
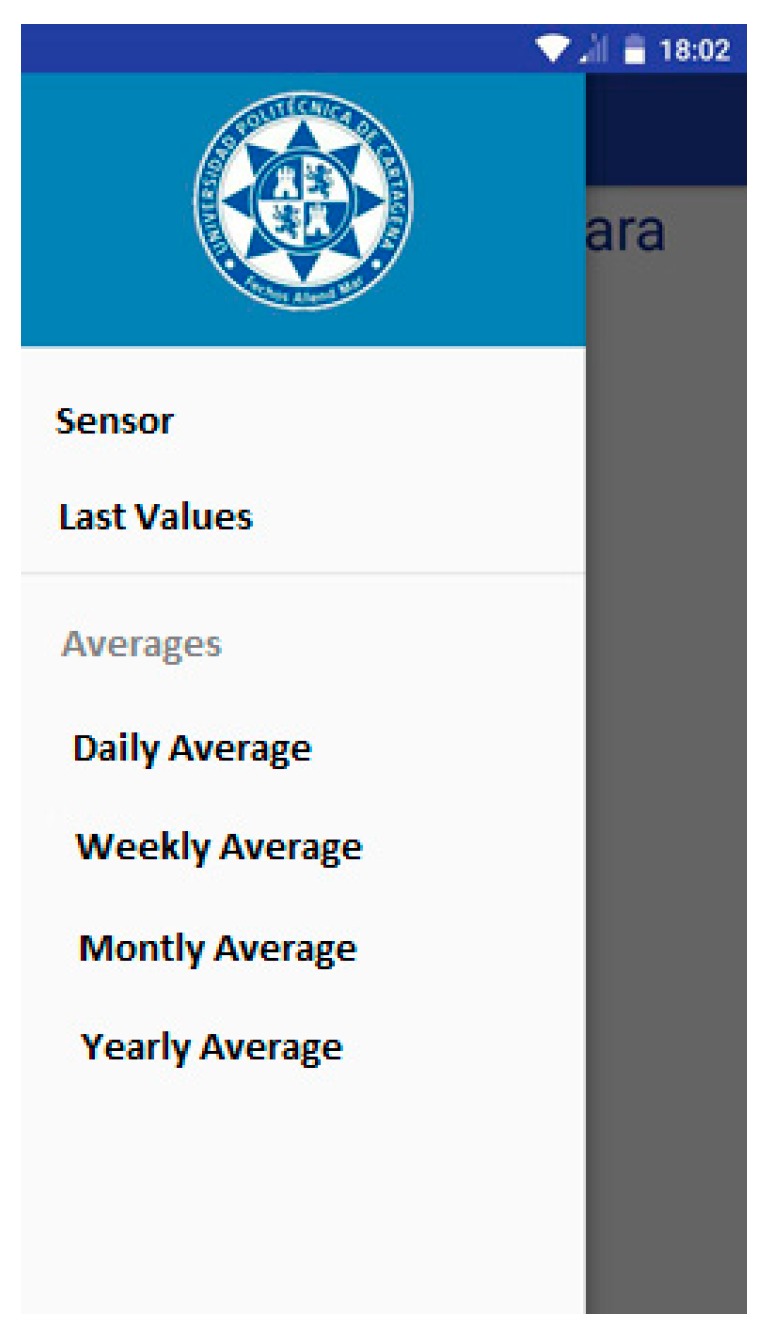
App statistical options.

The appearance of the main menu corresponds to the Technical University of Cartagena (UPCT) logo as well as a dropdown menu which lets us select the action we want the device to furnish is shown in Figure 15.

**Figure 16:**
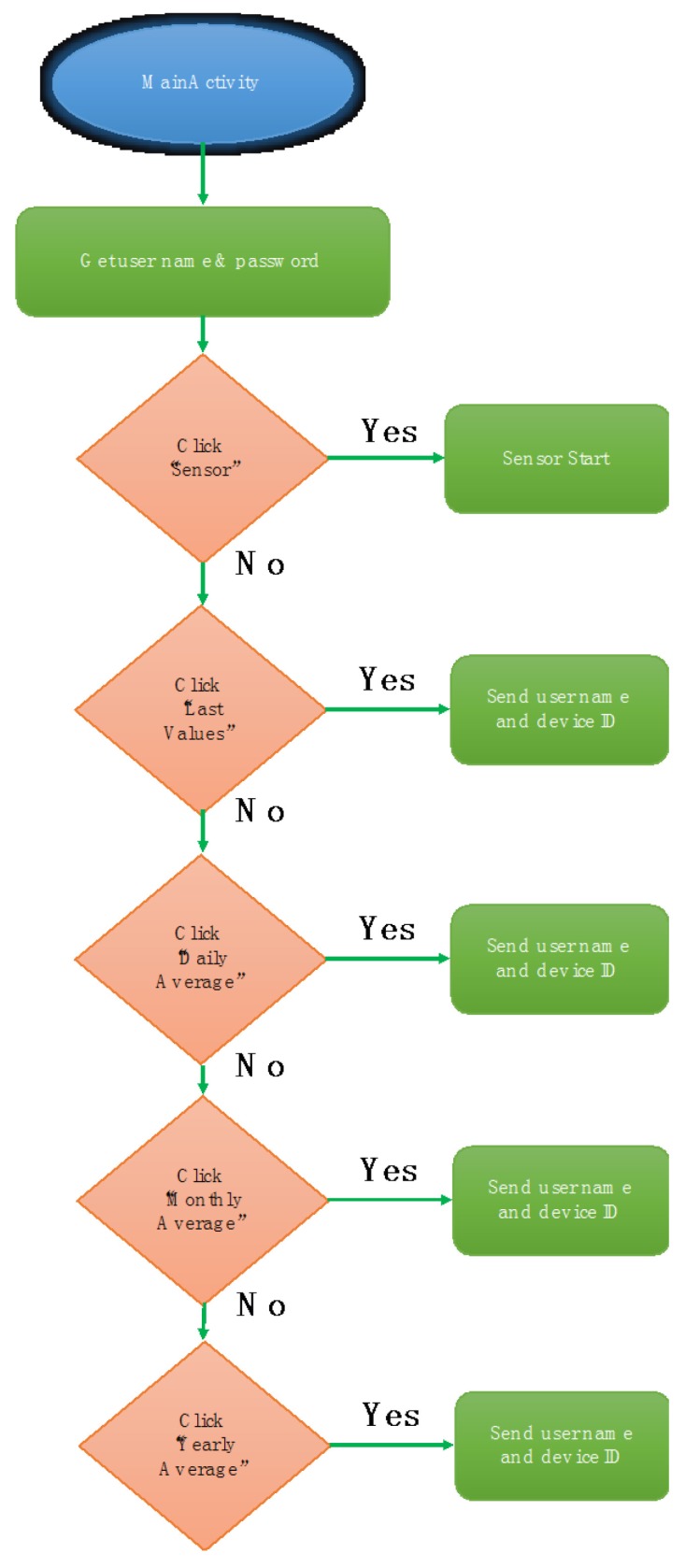
Main menu method flow-chart.

Each menu item is associated with a different activity. A particular case is the “Sensor” action, which shows GM detector (sensor) results in real time. The *MainActivity* allows a user who requires any stadistical action, for example, to download the monthly average radiation, to finish this action, and again access real time dose values in their smartphone. The goal is to provide an extra function to the main menu, which goes beyond an intermediary between activities. Finally, the function corresponding to the main activity is “MainActivity”, and is reflected in the following flowchart (Figure 16).

As shown in Figure 16, depending on the item of the dropdown menu which the user clicks, one action or another starts, and then a different activity begins. As previously illustrated in the “Intent” description, before starting a new activity, the user name and device number are obtained from “LoginActivity”.

##### Sensor (GM Detector)

**Figure 17:**
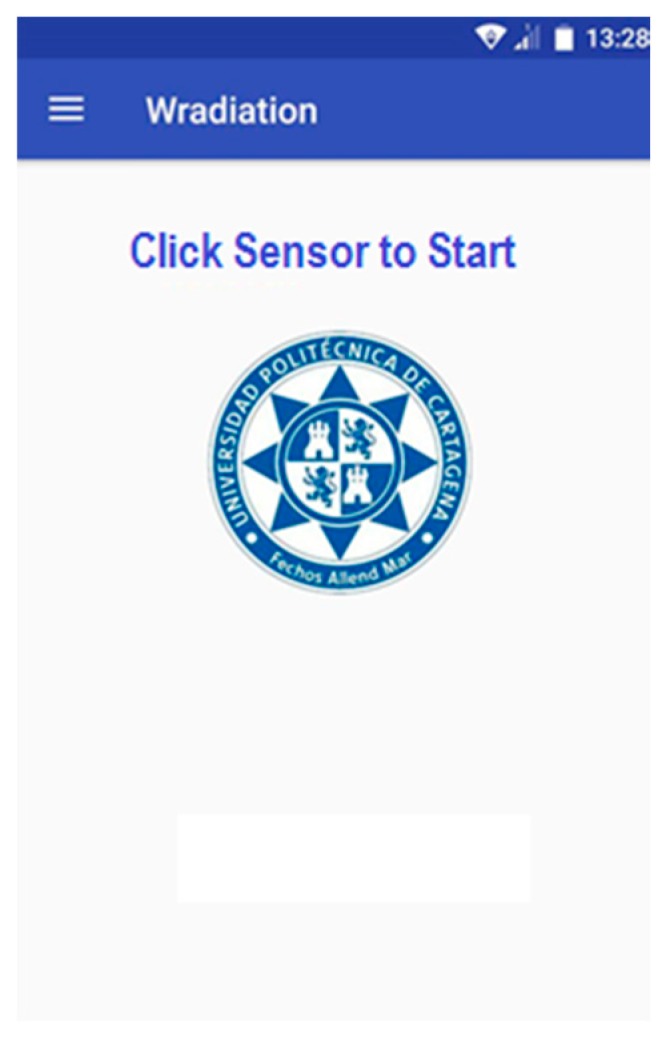
Main screen.

**Figure 18:**
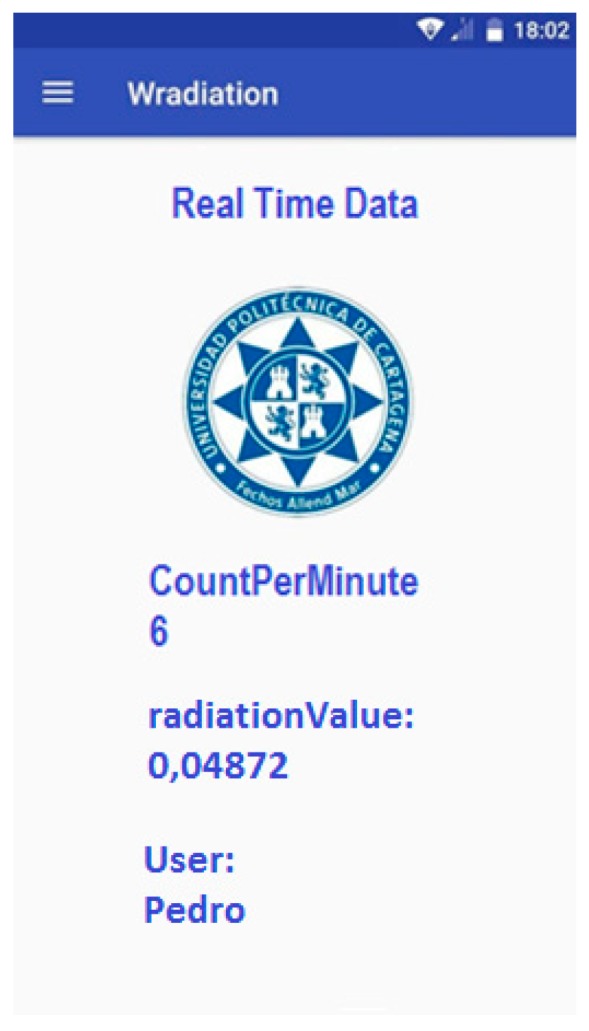
Dose values screen shot.

This activity is implemented in the “MainActivity”, sharing appearance and functionality with the dropdown main menu. The appearance depends on its state. If the “Sensor” icon has not been clicked by the user to start collecting and displaying values in real time, the following Figure 17 depicts the graphic user interface which appears. However, if the user clicks the “Sensor” icon, the window is Figure 18.

Values belong to counts per minute (CPM) and their corresponding radiation value, together with the user who has downloaded them, as shown in this view (Figure 18). These values are updated each second, collected by the GM detector. If the device turns off, the last value captured by the sensor is displayed. Note that the appearance changes (each second) once the first SQL query arrives to the database. The *Query* class directs this issue.

##### Showing Last Values

**Figure 19:**
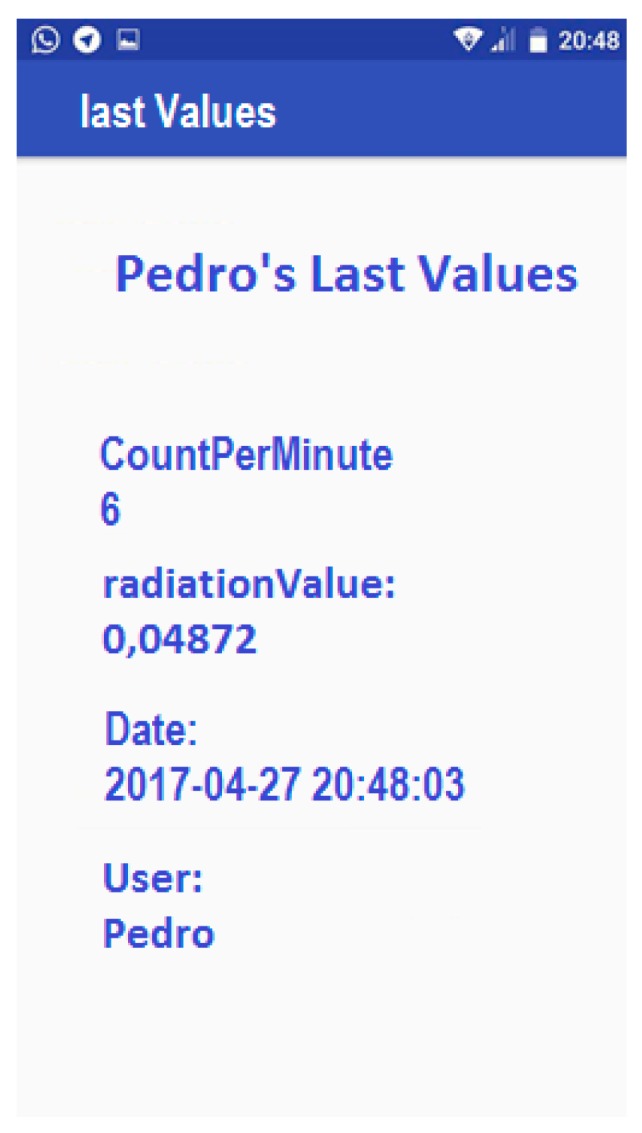
Last values screen shot.

This activity addresses the last counts per minute (and thus, radiation values) and the timestamp registered by the user device as well. The activity in charge of this function is Main2Activity. However, the class which leads to the connection to the database is Last class. This furnishes the required operations to obtain last dose values by means of SQL queries as shown in Figure 19.

##### Statistics

The appearance and functionality for the daily, weekly, monthly and annual average is the same. Only the SQL queries vary, taking into consideration the time period to compute. The statistic view shows the user name, the device number and the average value that is required to download from the remote database. The function is similar to the previous Last Values view. However, they are very different in the SQL query message. In detail, the activity addressed to the daily average is *Main4Activity* and the attachment to the database is carried out by the *diarilyaverage* class. Regarding the weekly average, it is accomplished by *Main5Activity* and the *weeklyaverage* class. The monthly average is run by *Main3Activity* and the *Middle* class. Finally, for the annual average, *Main6Activity*, together with the *MediaAnnual* class exhibit the annual statistics. These codes, together with the complete software here proposed, are available in [3].

## 4. Verification of the Device

To guarantee the reliability and traceability of radiation measurements, it is necessary to verify the appropriate operation of the device in hospital environments. The verification procedure was carried out in the Laboratory of Ionizing Radiation Metrology at the National Dosimetry Center (CND). The CND is the largest Personal Dosimetry Service in Spain, and one of the largest in Europe in terms of monthly reading volume. It has been authorized for this purpose by the Nuclear Safety Council. Likewise, the National Dosimetry Center has been authorized to act as a Radiological Protection Unit in radio diagnostic installations.

The CND has an Ionizing Radiation Metrology Laboratory, available to the public healthcare sector and private initiatives. The calibration laboratory was certified by the National Accreditation Agency (ENAC) for calibrations in ionizing radiation and radioactivity. The certification is also recognized by the European Organization for Accreditation of Laboratories (EA). The Laboratory performs calibration of instruments for measuring ionizing radiation, as well as irradiation of personal dosimeters (DTL, film dosimeters, etc.), at protection and diagnostic levels.

### 4.1. Verification Procedure

The verification performed on the system corresponds to the PT-03 procedure, which is characterized in the certification of instruments for measuring ionizing radiation under X-rays.

To furnish the procedure, the device was set-up in the Test Room without any external casing, taking the center of the GM tube as a reference point. In this room, the device was put in a vertical position, orienting the GM tube towards the X-ray source, so that the tube was parallel to the anode-cathode direction.

**Figure 20:**
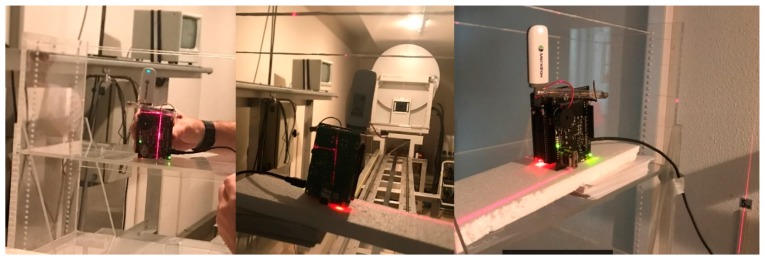
Device verification sequence.

In order to obtain the best measurement quality, the reference point was matched to the center of the radiation field. This was accurately marked by a laser, so the device was displaced to that point, as can be seen in the sequence of images in Figure 20.

The distance between the X-ray source and the reference point is 5 m. The appropriate radiation field diameter is 19 cm, capturing the radiation dose in the GM tube. The radiation qualities employed in the verification process are defined as N-80 and N-300 of the Narrow Spectrum ISO Series. The characteristics of these radiation qualities are broken down in Table 5, below. Note that *Kerma* is the sum of the kinetic energies of all charged particles set in motion by the radiation; its magnitude in the international system is the Gray, equivalent to a 1 Joule per Kilogram. On the other hand, the so-called half-value layer (HVL) is the layer thickness of copper reducing the intensity of radiation due to absorption and scattering phenomena by half (first half-value layer) and by four times (second half-value layer), respectively. As it will be observed in the next subsection, one of the test was carried out in the PET surroundings. In this device, the energy peak is 511 keV around, value that is out of the range of the N-300 quality. However, in usual medical tests where the radionuclide is inside the human body, the continuous spectrum generated is lower than the maximum peak. This fact is even greater in our case since we measure doses in the PET surroundings. Therefore, the N-300 quality is, a priori, appropriate to validate our device under these conditions. This also is in accordance with the CND standards. On the other hand, N-80 is the spectrum recommended for X-rays which usually varies from 20 to 125 keV; N-80 is selected as a central energy value.

Thus, the verification process performed is summarized in Table 6.

Table 6 exhibits the three tests performed on our device. They were defined by the CND according to the environment where our system will be deployed. Additionally, a thorough study should be carried out to enhance the GM measurement accuracy. For instance, in a future, a correction factor which reflects the difference between the response of the commercial GM in relation to the dose rate could be computed, to later program it in the Raspberry.

As regards the tests, firstly, an N-80 was implemented with a radiation range from 110 to 130 µGy/h, at an interval of 360 s. The second test was carried out with similar characteristics to the previous one, but increasing the radiation level from 180 to 210 µGy/h. Finally, an N-300 test was furnished with a radiation range from 350 to 400 µGy/h for an interval of 180 s.

In each test, the radiation values captured/sensed by the device were written down in Table 7. Later, these values were compared to the radiation values measured by the certified laboratory instrumentation at the same time interval. Thus, the relationship between the values captured by the device and those collected by the laboratory instrumentation results in an N_H_ verification factor. This factor must be multiplied by the radiation values measured by the device to obtain the true radiation values.

### 4.2. Verification Results

The verification results are shown Table 7 below.

Concerning the first N-80 test, the verification factor N_H_ is 3.37 with a deviation of ±0.20. The second N-80 test results in a N_H_ factor of 5.28 with a deviation of ±0.58. Finally, for the last N-300 test, the verification factor N_H_ is 10.2 with a deviation of ±1. Observing these results, we notice that as the radiation level increases, the radiation factor also grows considerably, having to multiply the radiation value of our device by 10.2 in the case of N-300 quality.

However, a last correction factor (which has been denoted as N_F_), motivated by the sampling period to which the GM tube is subject, must be considered. This factor adjusts our sampling times (1 s) to the one defined by the manufacturer (10-s blocks). In other words, by dividing the sampling period indicated by the manufacturer by 10, the sampling period under study is obtained, and then the CPMs are calculated. Therefore, the final verification equation is as follows:

Real Radiation Measurement = Captured Radiation Level *(N_H_/N_F_), (2)

where NF = 10.

This means that for the N-300 radiation quality, the standard deviation obtained in the verification process at the CND is the only one that computes.

## 5. Performance Evaluation in the Hospital

The main contribution of this work is to offer a step forward in radiation measurement systems and in particular, those affecting healthcare personnel. As a final issue in the development of the proposal, it was tested in a hospital environment under real conditions.

**Figure 21:**
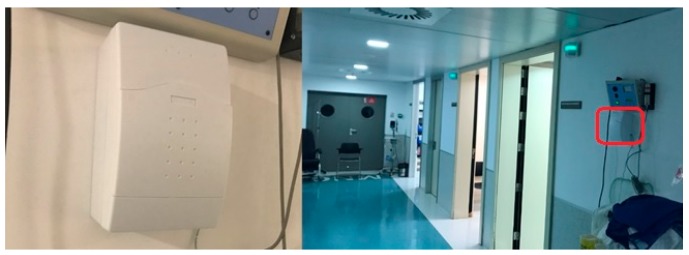
Device located in Positron Emission Tomography (PET) corridor.

Two devices were configured to be tested at St. Lucia University Hospital in Cartagena. A device was placed in the corridor adjacent to the Nuclear Medicine department (near the Positron Emission Tomography room, Figure 21), specifically, in the same place as the current area dosimeter. The device continuously measured the ambient radiation for 7.5 h. Its main purpose was to capture the radiation emitted by patients who walked by and stayed in this area.

**Figure 22:**
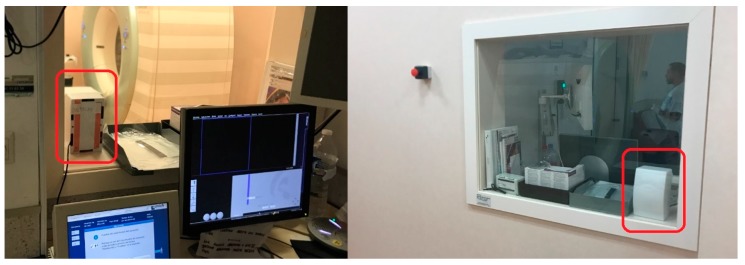
Device placed next to the CT room.

The second device was located in the CT area, specifically, in the room where the healthcare staff control the procedures (Figure 22). As in the previous case, this device measured environmental radiation. However, the radiation received by the healthcare staff was collected, measured, stored and computed in our databases and smartphone application.

An additional dosimeter was placed into our device cases for comparison purposes after the data collection. Those dosimeters were two small GM dosimeters from the Radiation Protection Department at Sta. Lucia University Hospital. The goal was to contrast the accumulated radiation in µSv/h measured by our devices with that obtained by Sta. Lucia dosimeters.

### Result Discussion

**Figure 23:**
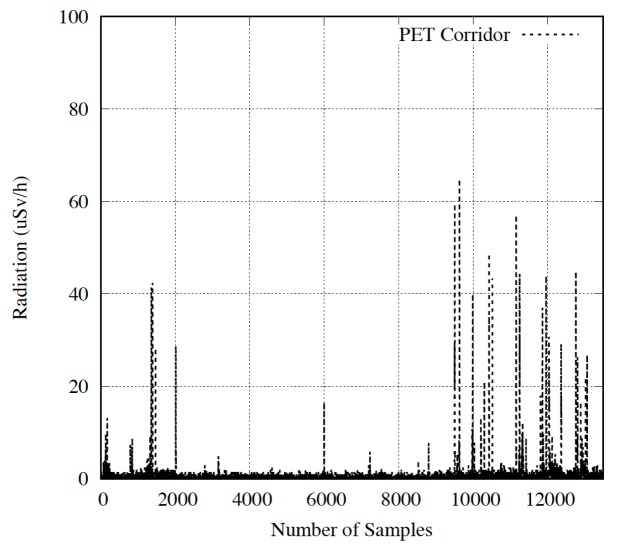
PET corridor results.

**Figure 24:**
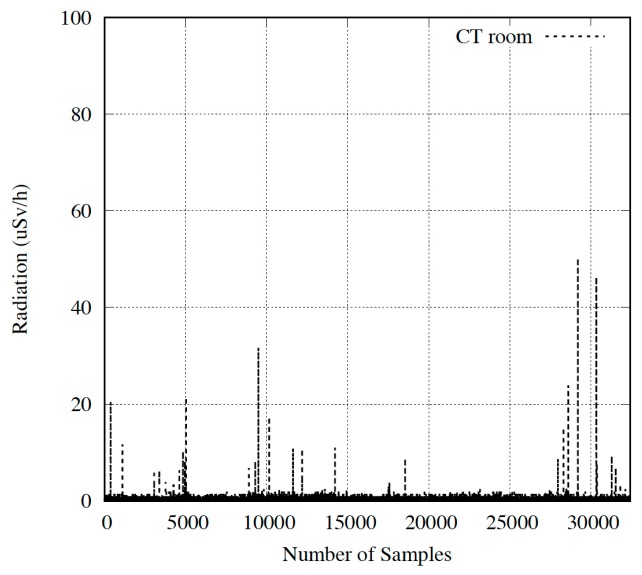
Results in room next to CT room.

The radiation values obtained by the devices can be observed in Figure 23 and Figure 24. Figure 23 shows doses captured in the corridor, while Figure 24 depicts results in the room next to CT. We will discuss the entire results, dividing them into two subsections depending on the placement of the devices.

#### Device Located in the Corridor

Before starting the tests, it was verified that the environmental radiation value captured by the device was similar to the one measured by the area dosimeter belonging to St. Lucia University Hospital. In detail, the latter obtained a dose range from 0.005 µSv/h to 0.5 µSv/h in a period of ten minutes. During the same interval, our device collected a radiation range from 0.0044 µSv/h to 0.4872 µSv/h.

As shown in Figure 23, we can observe that the radiation values increase to a maximum value (peak) and then they decrease gradually. As expected, a patient in treatment increments the activity of the device. Therefore, when values grow, it indicates that the patient is approaching the device. The maximum radiation value points out that the patient is closest to the device. Finally, as the patient moves away, the radiation values decrease.

Results were compared to the portable GM device at St. Lucia University Hospital. Our platform obtained an accumulative radiation of 2.46 µSv/h, compared to the 2.12 µSv/h of the hospital device during the time period evaluated. All in all, the viability and operation of our system as an area dosimeter is demonstrated, and validates the design and development proposed. Furthermore, these results can be displayed in real time, thanks to the app, or later analyzed, by dispatching data from the database (e.g., PC).

#### Device Located in the Room Next to CT System

Once installed and configured, the device was checked to observe if it captured radiation values when patients were scanned in real time. This task was carried out by two healthcare staff members, downloading and using the smartphone application, simultaneously. The result was that both received an accumulative dose of 0.45 µSv/h in their working day (eight hours).

Figure 24 shows the radiation values received by personnel during an 18 h period. As can be observed, the number of samples indicating a radiation level above the background value is lower than in the previous case. Therefore, the time dedicated to a complete CT scan is shorter than the radioactive pharmacological treatment received by a patient.

On the other hand, as in the corridor device, data obtained was compared to the portable GM device at St. Lucia University Hospital. Our platform obtained an accumulative radiation of 1.86 µSv/h in comparison with the value of 2.09 µSv/h measured by the hospital device during the time period evaluated, validating our system as a personnel dosimeter for specific areas. As in the previous case, these results can be monitored in real time or later, by transferring data from the database.

In both scenarios, it is noteworthy to mention that medical devices barely interfere in the communications of our devices due to the distances among them. This means that packet losses motivated by interferences are scarce, ensuring that data dispatched each second by the device are in turn received by the mobile application. Additionally, medium access mechanisms such as CSMA-CA (Carrier sense multiple access with collision avoidance) in WiFi support packet collision; the device transceiver senses the physical medium and, only when it is free of interferences, sends the packet including the collected dose.

## 6. Conclusions

In this paper, we propose the design and implementation of a low-cost, open-source, portable, COTS system to collect ionizing radiation (gamma particles) in real time, deriving (i) the average radiation in different temporal periods and (ii) the accumulated radiation received by medical personnel. The solution is offered by a device that contains a commercial Geiger-Muller detector and all the electronic boards required (hardware and software) to dispatch the dose values to a remote database. Healthcare staff will obtain information of the radiation received in their bodies through a mobile application, developed in Android, and connected to the database.

To validate the complete system, a twofold procedure was adopted. Firstly, it was verified in the CND Calibration Laboratory, an authorized and accredited center for this type of calibrations. Secondly, a test-bed was set up at Sta. Lucia University Hospital, deploying two devices in two potential radiation areas and comparing measurements with other validated dosimeters placed next to our devices. Results show that this solution is able to accurately measure the radiation doses received by two heathcare workers in real time, and generate statistics for later studying and analyzing. Finally, the system proposed appropriately operates as a general purpose area dosimeter.

## Figures and Tables

**Table 1:** Dose level ranged by exposition category.

Healthcare Professional Category	Effective Dose	Eye Lense Dose	Extremity Dose
Exposed worker A classification	>6 mSv/year	>45 mSv/year	>150 mSv/year
Exposed worker B classification	<6 mSv/year	<45 mSv/year	<150 mSv/year
Non-exposed workers	<1 mSv/year	<15 mSv/year	<50 mSv/year

**Table 2:** Percentage of healthcare workers exceeding the annual dose limit according to their job type.

Job Type	Doctor	Medical Assistant	Operator	Other
Outpatient clinic Radiology	0.0%	0.40%	0.0%	0.0%
Hospital Radiology	2.7%	1.40%	0.5%	0.4%
Vascular Radiology	24.1%	5.90%	2.9%	1.5%
Radiotherapy	0.1%	2.60%	0.4%	0.7%
Nuclear Medicine	1.7%	50.70%	36.6%	13.0%
Interventional	2.2%	0.40%	0.0%	0.1%
Others	0.6%	0.30%	0.0%	0.0%

**Table 3:** Radiation range for personal dosimeter.

Dosimeter	Range
Film and TLD dosimeters	100 µSv–10 Sv
OSL and RPL dosimeters	10 µSv–10 Sv
Self-reading pocket dosimeters	50 µSv–0.2 Sv
Electronic Personal dosimeter	0.1 µSv–10 Sv

**Table 4:** Comparison among different dosimeters including our system.

Device	Real Time	Reusable	Low-Cost	Internet	Database	Reliability	Smartphone App	Open Source
TLD Dosimeter	No	Yes	Yes	No	No	Yes	No	No
Film Dosimeter	No	No	N/A	No	No	Yes	No	No
RPLGD	No	Yes	N/A	No	No	Yes	No	No
OSL Dosimeter	No	Yes	N/A	No	No	Yes	No	No
Personal Elec. Dosimeter	Yes	Yes	No	No	No	Yes	No	No
Pocket Dosimeter	Yes	Yes	Yes	No	No	No	No	No
Ionization Chamber	Yes	Yes	No	No	N/A	Yes	No	No
Proportional Counter	Yes	Yes	No	No	N/A	Yes	No	No
Geiger-Müller Counter	Yes	Yes	No	No	Yes	Yes	No	No
Scintillator Counter	Yes	Yes	No	No	N/A	Yes	No	No
Semiconductors detector	Yes	Yes	N/A	No	N/A	Yes	No	No
Instadose-Mirion	Yes	Yes	N/A	No	Yes	N/A	Yes	No
Dosicard-Canberra	Yes	Yes	N/A	No	Yes	N/A	No	No
PROPOSED SYSTEM	Yes	Yes	Yes	Yes	Yes	Yes	Yes	Yes

**Table 5:** Main features for N-80 and N-300 Qualities.

Quality Code	Voltage (Kv)	E_avg_ (keV)	1st HVL (mm Cu)	2nd HVL (mm Cu)	Kerma (Air) (µGy/min) Minimum	Kerma (Air) (µGy/min) Maximum
N-80	80	65	0.05776	0.619	1.9	1000
N-300	300	250	6.28	6.29	2.9	450

**Table 6:** Verification procedure for N-80 and N-300 Qualities.

Quality Code	System Scale	Kerma Air Rate (µGy/h)	Radiation Time
N-80	100	110–130	360
N-80	100	180–210	360
N-300	100	350–400	180

**Table 7:** Verification results for our proposal.

Quality Code	System Scale	Kerma Air Rate (µGy/h)	Radiation Time	N_H_
N-80	100	110–130	360	3.37 ± 0.20
N-80	100	180–210	360	5.28 ± 0.58
N-300	100	350–400	180	10.2 ± 1.0
